# Factors associated with oral anticoagulant prescription status among patients with a new diagnosis of atrial fibrillation

**DOI:** 10.1002/clc.24077

**Published:** 2023-07-04

**Authors:** Evan Manning, Kelley Burns, Melissa Laurie, Luke Patten, Michael Ho, Amneet Sandhu

**Affiliations:** ^1^ Internal Medicine Residency Training Program University of Colorado Anschutz Medical Campus Aurora Colorado USA; ^2^ Data Science to Patient Value (D2V) University of Colorado Anschutz Medical Campus Aurora Colorado USA; ^3^ US Health Economics & Outcomes Research Bristol Myers Squibb Lawrenceville New Jersey USA; ^4^ Center for Innovative Design & Analysis University of Colorado Anschutz Medical Campus Aurora Colorado USA; ^5^ Deparment of Medicine University of Colorado Anschutz Medical Campus Aurora Colorado USA

**Keywords:** anticoagulant therapy, atrial fibrillation, quality of Care, stroke prevention

## Abstract

**Background:**

Atrial fibrillation (AF) is the most common sustained arrhythmia in adults and increases stroke risk. Treatment with oral anticoagulants (OACs) may reduce this risk however many patients do not receive OAC therapy. This study aimed to use electronic health record data to identify newly diagnosed AF patients at high risk for stroke and not anticoagulated as well as factors associated with OAC prescription.

**Hypothesis:**

Timely prescription of OACs among patients with newly diagnosed AF is poor.

**Methods:**

We performed a retrospective study of patients with a new diagnosis of AF. We assessed stroke risk with the CHA_2_DS_2_‐VASc score. The primary outcome was prescription of an OAC within 6 months following diagnosis. We used logistic regression to see how the odds of being prescribed an OAC differs for 17 independent variables.

**Results:**

We identified 18 404 patients with a new diagnosis of AF. Among patients at high risk for stroke, 41.3% received an OAC prescription within 6 months. Male sex, Caucasian compared to African American race, stroke, obesity, congestive heart failure, vascular disorder, current antiplatelet, beta blocker, or calcium channel blocker prescription, and increasing CHA_2_DS_2_‐VASc score were positively associated with receiving an OAC. Whereas anemia, renal dysfunction, liver dysfunction, antiarrhythmic drug use and increasing HAS‐BLED score were negatively associated.

**Conclusions:**

Most newly diagnosed AF patients at high stroke risk do not receive an OAC prescription in the first 6 months following diagnosis. Our analysis suggests that patient sex, race, comorbidities, and additional prescriptions are associated with rates of OAC prescribing.

## Graphical abstract

Oral anticoagulant (OAC) prescribing rates are poor in the first 6 months following a new diagnosis of atrial fibrillation (AF). Patient characteristics, including sex, race, comorbidities, bleeding risk, and current prescriptions may influence OAC prescribing. Understanding these associations could improve quality of care among patients with AF at risk of stroke.

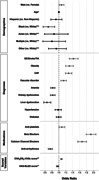

## 1 INTRODUCTION

Atrial fibrillation (AF) is the most common dysrhythmia in the United States and is associated with an increased risk of thromboembolic events including stroke.^1^ Treatment with oral anticoagulants (OACs) may mitigate stroke risk however OAC use is known to be poor among patients with AF and who are eligible for this therapy with recent analyses of both regional and national databases suggesting an overall prescription rate of approximately 60%.^2^, ^3^ With the annual cost of stroke in the United States estimated at over 36 billion dollars, there is a public health interest in improving OAC prescription rates in this population.^4^

Previous studies have explored prescribing associations by prescriber specialty, patient characteristics such as sex and race, and social determinants of health such as income and the built environment. For example, individuals seen by a cardiologist after a diagnosis of AF are more likely to receive appropriate anticoagulation of any form and are more likely to receive a direct OAC (DOAC) rather than a Vitamin K Antagonist (VKA) compared to those seen only by a primary care physician.^5^ A study in the Veterans Affairs population determined that the odds of receiving an appropriate OAC prescription was lower in both Asian and Black populations compared to Whites and the odds of receiving a DOAC compared to a VKA was likewise lower among minorities.^6^ While evaluations of the built environment via the neighborhood deprivation index have not demonstrated a difference in overall OAC prescriptions, a higher deprivation index is associated with a lower likelihood of DOAC prescription.^7^ Additionally, patient insurance has been identified as a predictive factor of VKA versus DOAC prescription.^8^

In terms of patient factors associated with OAC prescriptions, it is well‐established that increasing age and increasing HAS‐BLED scores are associated with fewer OAC prescriptions.^9^ Studies in the United Kingdom and among the elderly have identified increasing Charlson Comorbidity Index as negatively associated with OAC prescriptions.^10^, ^11^ However, the influence of specific clinical factors such as individual comorbid health conditions and concurrent prescriptions is less studied with prior evaluations limited in geographic scope and occurring before the widespread availability of DOAC in more recent years.^12^ We aimed to utilize electronic health record (EHR) data to (1) identify rates of OAC prescriptions among patients with newly diagnosed AF and at high risk for stroke and (2) evaluate factors associated with OAC prescription.

## 2 METHODS

### 2.1 Setting

The University of Colorado Health System (UCHealth) is a large, integrated healthcare network comprised of an academic medical center and 11 community‐based hospitals. Data from UCHealth hospitals are captured through a shared EHR system (Epic).

### 2.2 Study population

EHR data were utilized to construct a retrospective cohort of patients with newly diagnosed AF between January 1, 2013 and December 31, 2018. Patients were identified using ICD‐9 and ICD‐10 codes. An 18‐month baseline period before diagnosis was used to screen patients for prior use of the UCHealth system, prior AF diagnosis, and prior OAC use. Those without an AF diagnosis in this period were considered to have a new diagnosis. Those without a previous encounter, with an existing prescription for an OAC, or with CHA_2_DS_2_‐VASc score < 2 were excluded. Additional exclusion criteria were pregnancy, valvular disease, and death during the index encounter.

### 2.3 Study variables

The primary outcome was prescription of an OAC during the first 6 months following AF diagnosis. The 6‐month period was selected to provide a reasonable timeframe for outpatient medication initiation and in consistency with other studies that use similar periods to define new AF ranging 90 days to 6 months.^4^, ^6^, ^13^ OAC included prescriptions for VKA and DOACs. Secondary analyses determined odds of OAC prescription with respect to 17 independent clinically relevant variables.

### 2.4 Statistical analysis

Logistic regression was performed to compare the odds of OAC prescription with 17 predefined clinical variables. For noncategorical variables, odds were reported per 10‐year increase in age and per 1‐unit increase in CHA_2_DS_2_‐VASc or HAS‐BLED score. Tukey‐adjusted pairwise comparisons were calculated for each race compared to White, to determine race‐based odds ratios.

## 3 RESULTS

**Figure 1:**
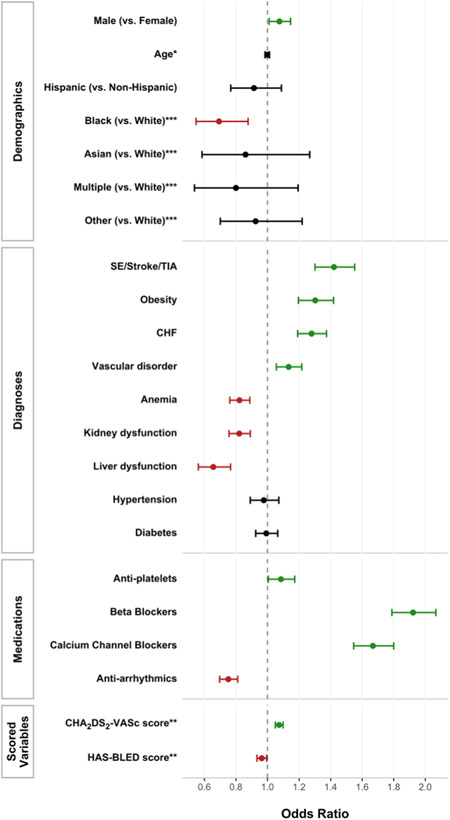
Forest plot of regression results (with odds ratio and 95% C.I.) for all factors. *for a 10‐year increase in age. **Tukey‐adjusted pairwise comparisons are shown for each race compared to white, where all other pairwise comparisons for race were statistically insignificant. ***for a 1‐unit increase in the scored variables.

Of 18 404 patients (53% male, median age 73.9 years) who met inclusion criteria, 7604 (41.3%) received an OAC prescription within 6 months of AF diagnosis. 35.8% of all OAC prescriptions during the study period were for DOACs. The proportion of OAC prescriptions for DOACs increased yearly from 10.5% in 2013 to 53.7% in 2019. Conversely, the proportion of OAC prescriptions for VKAs decreased from 89.5% in 2013 to 46.3% in 2019. Male sex (OR 1.076, [95% CI 1.011−1.146]), Caucasian compared to African American race (1.443, [1.140−1.828]), history of stroke (1.421, [1.301−1.552]), obesity (1.302, [1.195−1.418]), congestive heart failure (1.279, [1.190−1.374]), or vascular disorder (1.134, [1.056−1.217]), current use of antiplatelet agent (1.085, [1.005−1.172]), beta blocker (1.923, [1.788−2.067]), or calcium channel blocker (1.668, [1.546−1.800]), and CHA_2_DS_2_‐VASc score (1.074, [1.050−1.099]) were positively associated with OAC prescription. History of anemia (0.822, [0.761‐0.888]), kidney dysfunction (0.821, [0.756−0.891]), or liver dysfunction (0.657, [0.562−0.766]), current use of antiarrhythmic drug (AAD; 0.752, [0.697−0.811]) and HAS‐BLED score (0.964, [0.933−0.995]) were negatively associated. Logistic regression results are summarized in Figure 1.

## 4 DISCUSSION

We found most patients with a new AF diagnosis and elevated CHA_2_DS_2_‐VASc are (1) not prescribed an OAC within the first 6 months after diagnosis and (2) odds of receipt of a timely prescription are independently associated with patient sex, race, comorbid medical conditions, current prescriptions, and calculated bleeding risk. While causal factors for different rates of OAC prescribing remain to be determined, these associations may provide some insight into OAC prescribing decisions. Unsurprisingly, estimated bleeding risk, comorbid conditions predisposing to bleeding such as anemia and liver dysfunction, and renal dysfunction which limits the use of certain anticoagulants are associated with fewer OAC prescriptions. Analogously, additional indications for anticoagulation such as prior stroke or vascular disorder increase OAC prescriptions. Other results are less intuitive. The use of AADs would be expected to correlate with OAC use however it is negatively associated in this study. We postulate that AADs may influence OAC prescriptions among patients with lower CHA_2_DS_2_‐VASc scores and paroxysmal AF, particularly those who have a low arrhythmia burden. We hypothesize that the use of antiarrhythmic therapy suggests an intent to pursue a rhythm control strategy, thereby influencing some clinician decisions to discontinue or forgo anticoagulation. However, this is still contradictory to current guidelines in AF management. Antiplatelet agent use was positively associated with OAC prescription in this work which is contrary to prior studies.^14^ We hypothesize that this inconsistency is, in part, due to differences in study populations where providers and patients in studies focused on the elderly may place greater weight on the risk of bleeding in their discussions about whether to be on combination antiplatelet and anticoagulant therapy compared to our study which included all patients over the age of 18.

The salient finding of this work is less than half of those eligible for long‐term OAC with newly diagnosed AF receive timely therapy. As evidenced by our findings, patient‐level factors are associated with increased or decreased OAC prescriptions and may represent opportunities for targeted study and intervention to reduce the risk of stroke in the greater population. All these associated factors represent possible future directions of study.

There are several limitations to our study. First, this study is naturally limited by its observational design. Second, EHR data is limited by the accuracy of coding of patient demographics, visit diagnoses, and pharmaceutical information. Third, data were collected from 2013 to 2018 and may not reflect current prescribing trends, particularly for DOACs, as VKAs were the predominant OAC prescribed throughout this study with DOACs increasing in prevalence every year. Finally, this analysis is limited to a single health system where data could be influenced by regional practice and prescribing patterns. Additionally, prescriber specialty, which has been previously shown to affect OAC prescribing, and reasons for lack of OAC prescription were not captured nor were the intent of this work but present future direction for study.^5^

## 5 CONCLUSIONS

Our work found that OAC prescription rates are poor, in the first 6 months, among those with newly diagnosed AF. Patient characteristics, including sex, race, comorbidities, bleeding risk, and current prescriptions may influence OAC prescribing. Understanding these associations could improve quality of care among patients with AF at risk of stroke.

## CONFLICTS OF INTEREST STATEMENT

Melissa Laurie is a Bristol Myers Squibb employee. The remaining authors declare no conflict of interest.

## Data Availability

The data that support the findings of this study are available from the corresponding author upon reasonable request.
